# Putative antigenic proteins identified by comparative and subtractive reverse vaccinology in necrotic enteritis-causing *Clostridium perfringens* isolated from broiler chickens

**DOI:** 10.1186/s12864-021-08216-7

**Published:** 2021-12-13

**Authors:** Ilhem Meniaï, Alexandre Thibodeau, Sylvain Quessy, Valeria R. Parreira, Philippe Fravalo, Guy Beauchamp, Marie-Lou Gaucher

**Affiliations:** 1 Département de Pathologie et Microbiologie, Faculté de médecine vétérinaire, Université de Montréal, 3200 Sicotte, St-Hyacinthe, QC J2S 2M2 Canada; 2 Swine and Poultry Infectious Diseases Research Centre (CRIPA), Faculté de médecine vétérinaire, Université de Montréal, 3200 Sicotte, St-Hyacinthe, QC J2S 2M2 Canada; 3 Department of Food Science, University of Guelph, Guelph, ON N1G 2W1 Canada; 4 Faculté de Médecine Vétérinaire, Université de Montréal, Saint-Hyacinthe, QC J2S 2M2 Canada

**Keywords:** Broiler chickens, Necrotic enteritis, *Clostridium perfringens*, Comparative subtractive reverse vaccinology, Candidate proteins, Vaccine

## Abstract

**Background:**

Avian necrotic enteritis (NE) caused by *Clostridium perfringens* is a disease with a major economic impact, generating losses estimated to 6 billion of dollars annually for the poultry industry worldwide. The incidence of the disease is particularly on the rise in broiler chicken flocks eliminating the preventive use of antibiotics. To date, no alternative allows for the efficient prevention of NE and a control of the disease using a vaccinal strategy would be mostly prized. For this purpose, comparative and subtractive reverse vaccinology identifying putative immunogenic bacterial surface proteins is one of the most promising approaches.

**Results:**

A comparative genomic study was performed on 16 *C. perfringens* strains isolated from healthy broiler chickens and from broilers affected with necrotic enteritis. Results showed that the analyzed genomes were composed of 155,700 distinct proteins from which 13% were identified as extracellular, 65% as cytoplasmic and 22% as part of the bacterial membrane. The evaluation of the immunogenicity of these proteins was determined using the prediction software VaxiJen®.

**Conclusions:**

For the most part, proteins with the highest scores were associated with an extracellular localisation. For all the proteins analyzed, the combination of both the immunogenicity score and the localisation prediction led to the selection of 12 candidate proteins that were mostly annotated as hypothetical proteins. We describe 6 potential candidates of higher interest due to their antigenic potential, their extracellular localisation, and their possible role in virulence of *C. perfringens*.

## Background

After the 2014 report published by the World Health Organization (WHO) highlighting the serious issue of antimicrobial resistance and its threat to public health, various action plans were developed and adopted all over the world in order to control and regulate antimicrobial use [1]. In Canada, an action plan was launched aiming to create and reinforce surveillance of antimicrobial resistance in animals and humans, in order to (i) promote responsible use of antimicrobials in both veterinary and human medicine, (ii) create collaborations between industrial and agricultural partners, and (iii) promote research and innovation at the national and international levels [2].

As part of the initiatives undertaken by the food-producing animal industries in Canada, the Chicken Farmers of Canada (CFC) is currently implementing its national antimicrobial use reduction strategy (AMU strategy) which main objective consists in a gradual elimination of the preventive use of antimicrobials under the first three categories of antibiotics defined as important for human health by Health Canada (Table 1) [3].

**Table 1:** Categories of important antimicrobials used in poultry production in Canada and their removal date according to the CFC’s AMU strategy.

Category	Examples of antimicrobials	Usage in poultry	Removal date
I	Fluoroquinolones	Treatment of bacterial infections	2015
	Cephalosporins 3rd generation		
II	Aminoglycosides	Prevention of neonatal infections in chicks	2018
	Lincosamides-aminocyclitols		
	Macrolides	Treatment of bacterial infections	
	Penicillins		
	Streptogramins	Prevention of enteric diseases	
	Trimethoprim-sulfamides	Treatment of pathogenic avian *E. coli* infections	
III	Bacitracin	Prevention of enteric diseases	To be determined
	Tetracyclines	Treatment of bacterial infections	
IV	Flavophospholipids	Growth promotion	
	Ionophores	Coccidiosis control	

In 2006, the European Union countries banned the use of antibiotic growth promoters, which, based on the results of field studies conducted in Canada, will most likely be associated with the re-emergence of necrotic enteritis (NE) that had been kept under control with the historical routine use of antibiotics [4, 5]. Different alternatives to the use of antibiotic growth promoters such as the modification of the diet by the addition of probiotics, prebiotics, and essential oils, but also phage therapy and vaccines have been tested for the control of NE on broiler chicken farms [6]. To date, none of these approaches have shown promise in the successful replacement of antimicrobials.

It is well-known that different factors such as coccidiosis, the immune status of the birds, and the diet composition can predispose broiler chickens to NE [7], despite the fact that some strains of *Clostridium perfringens* have been identified as a key element for disease occurrence as well [8].

*C. perfringens* is a bacterium ubiquitously found in the environment, as well as in the gut microbiota of all warm-blooded animals [9]. Based on the carriage of various toxin genes, this bacterial species has been classified into 7 toxinotypes, from which *C. perfringens* type G is recognized to cause NE [9]. Up to now, virulence of type G strains has mainly been associated with the necrotic enteritis B toxin (NetB), but more recent studies have suggested possible different roles for other virulence factors in the pathogenicity of the bacterium [8]. The *netB* gene was found on the largest of three pathogenicity loci identified in virulent *C. perfringens*, NELoc-1 [10]. From a mutant *C. perfringens* strain, a study showed that in the absence of other NELoc-1 genes, *netB* restored virulence only partially, suggesting that other genes, as well as the regulation of *netB* expression, may play a role in the virulence of *C. perfringens* [11]. Lepp and collaborators also identified another locus, VR-10B, which is a variable region found in pathogenic strains of avian *C. perfringens*. This locus has been described as a facilitator of adhesion to extracellular matrix molecules by encoding proteins involved in gut colonization, a crucial step in NE pathogenesis [8, 12]. As proposed earlier by Prescott et al. [7], NE pathogenesis can be broken down in various steps of which colonization of NE-causing *C. perfringens* would be preceded by the displacement of the resident *C. perfringens* population with the help of bacteriocins. Indeed, it has been shown that the gene encoding the perfrin bacteriocin was found in 90% of NE-causing *C. perfringens* strains. Therefore, perfrin-bacteriocin was proposed to be an important factor contributing to the competitiveness of virulent strains of avian *C. perfringens* [8, 13]. The degradation of the mucous layer by degrading enzymes for which the encoding genes have also been identified in the pathogenicity loci of NE-causing *C. perfringens* would allow for the initial colonization of virulent *C. perfringens* [7]. The pore-forming toxin NetB, by damaging the intestinal epithelial cells and exposing the host extracellular matrix proteins would foster the deeper intestinal colonization by virulent *C. perfringens* through the action of adhesins from which some are known to be encoded by genetic elements being part of the typical genetic signature of NE-causing *C. perfringens* [7]. The variety of virulence factors described so far and the potential for the discovery of new factors hidden in the *C. perfringens* genetic baggage highlight the need for continuous research of bacterial attributes that could be targeted for the development of innovative methods for controlling avian necrotic enteritis.

In recent years, the ease of accessibility to high-throughput sequencing technologies and computational methods in genomics allowed for a deeper analysis of bacterial genomic data in a wide array of applications. In the context of vaccine development, reverse vaccinology has been introduced as a new and highly competitive approach for the identification of putative bacterial antigens. Reverse vaccinology allows for a rapid assessment of the antigenic potential of candidate proteins in pathogenic microorganisms by investigating their genome composition, and more importantly, by describing the surface proteome of the screened microorganisms. Based on an *in silico* identification of proteins, the rapid screening of potential candidate antigens allows for a more efficient identification of putative vaccine candidates. Moreover, the comparative and subtractive approach of the reverse vaccinology method allows for the selection of vaccine candidates unique to virulent strains of bacteria by assessing the dissimilarities between disease-causing and non-disease-causing strains of a specific bacterial species. This method was used in various studies and the first approved vaccine developed using a comparative reverse vaccinology approach, MenB, was commercialized in 2012 [14–16].

Using comparative and subtractive reverse vaccinology, the aim of this study was to identify putative antigenic proteins unique to NE-causing *C. perfringens* that could serve in the development of an effective control strategy against avian NE.

## Material and methods

### *Clostridium perfringens* strains selection

For the comparative and subtractive reverse vaccinology approach, 16 *C. perfringens* sequenced strains were selected based on previous characterization data pertaining to the toxinotype, PFGE profile, the type IV pilus profile, the toxinotype and the ability to cause NE (see Table 2 [5, 6]. For the purpose of the current study, the presence of the *cnaA* gene was verified by PCR according to Wade *et al.* [17]. Briefly, each PCR reaction was conduct with 3 mM of MgCl_2_, 0.4 mM of dNTPs, 1 U of Taq polymerase (Biobasic, TAQ DNA polymerase High purity, cat. # HTD0078) and 0.5 μM of each primer. PCR conditions were as follows: 95 °C for 2 min, 35 cycles at 95 °C for 20s, 50 °C for 20s and 72 °C for 30s, and a final extension at 72 °C for 5 min. From the 16 strains selected, 6 virulent and 10 commensal *C. perfringens* were analyzed. These strains were sequenced, and their genomes were screened for the identification of putative antigenic proteins using a comparative and subtractive reverse vaccinology approach.

**Table 2:** Strain profiles and genome assembly statistics of virulent and commensal *C. perfringens* strains analyzed in this study.

Strain type	Strain identification	***netB*** status	***cpb2*** status	***tpeL*** status	***cnaA*** status	PFGE profile	Type IV pilus profile	Total genome length (Mb)	No. contigs	GC content%	No. CDS
Virulent	MLG_2313	+	+	–	+	V3	9	3.53	113	27.99	3204
	MLG_2514	–	–	–	+	V5	5	2.95	722	29.01	2539
	MLG_1202	+	–	–	+	V5	8	3.73	195	27.97	3471
	MLG_2915	+	–	–	+	V5	10	3.08	672	28.80	2669
	MLG_7814	–	–	–	+	V6	5	3.66	172	28.02	3415
	MLG_7820	+	–	–	+	V7	5	3.54	286	28.17	3262
	**Average**							**3.42**	**360**	**28.33**	**3093**
Commensal	MLG_5806	–	–	–	–	C1	6	3.32	25	28.12	2955
	MLG_4206	–	+	–	–	C2	2	3.39	80	28.04	3025
	MLG_3406	–	+	–	–	C2	7	3.39	82	28.05	3015
	MLG_5213	+	+	–	+	C3	6	3.51	122	28.00	3196
	MLG_0612	+	+	–	+	C3	6	3.51	91	27.98	3203
	MLG_3119	–	–	–	–	C1	7	3.43	41	28.04	3044
	MLG_2919	–	–	–	–	C1	20	3.43	34	28.04	3045
	MLG_2719	–	–	–	–	C1	21	3.43	32	28.03	3044
	MLG_2019	–	–	–	–	C2	3	3.22	88	28.18	2865
	MLG_1619	–	–	–	–	C3	22	3.26	69	28.17	2889
	**Average**							**3.39**	**66**	**28.07**	**3028**

### Culture of *Clostridium perfringens* strains

Selected strains were grown overnight on 5% sheep blood agar plates (Oxoid, Nepean, ON, Canada) at 37 °C under anaerobic conditions (AnaeroGen Gas Generating System, Oxoid, Nepean, ON, Canada). The MiSeq WGS Sequencing Method for *Clostridium perfringens* (#17–066474) used by the Agriculture and Food Laboratory of the University of Guelph was used and is described below.

### DNA extraction

DNA was extracted from the pure cell cultures using the DNeasy Blood and Tissue Kit (Qiagen) following the manufacturer’s protocol for Purification of Total DNA from Gram-positive Bacteria. Extracted DNA was eluted in 100 μL AE buffer, quantified using a NanoDrop ND-2000 UV Vis ND-2000 Spectrophotometer (Thermo Fisher Scientific) and a Qubit® Fluorometer with a Qubit® dsDNA BR Assay Kit (Thermo Fisher Scientific), and stored at −20 °C until further analysis.

### Sequencing library preparation

Whole genome sequencing libraries were prepared using Nextera™ XT DNA Library Prep kit following the procedures described in the Nextera™ XT DNA Sample Preparation Guide (Illumina, Doc# 15031942). Briefly, approximately 1–2 ng of genomic DNA was fragmented using Nextera transposome and tagged with adapter sequences. The adapter-tagged DNA was then amplified using a limited-cycle PCR program to add index adapters and sequences required for sequence cluster generation. The PCR thermal cycling conditions were: 72 °C for 3 min and then 95 °C for 30 s; 12 cycles of 95 °C for 10 s, 55 °C for 30 s and 72 °C for 30 s, followed by 72 °C for 5 min and then holding at 10 °C using a GeneAmp PCR System 9700 thermal cycler (Life Technologies). The amplified libraries were purified using Sample Purification Beads to remove free primers. The quality and quantity of the purified libraries were assessed by a Bioanalyser 2100 with Agilent High Sensitivity DNA Assay Kit (Agilent Technologies). DNA concentrations were also measured using a Qubit® Fluorometer and a Qubit® dsDNA BR Assay Kit (Thermo Fisher Scientific). The purified libraries were then normalized and combined in equal molar ratios based on their DNA concentrations; the pooled fragments were used for sequencing.

### DNA sequencing using the MiSeq system

The pooled libraries were denatured with NaOH and diluted with hybridization buffer prior to sequencing. PhiX (Illumina) was included at a 1% level to serve as an internal control. Sequencing was conducted using a MiSeq sequencer with a MiSeq v2 reagent kit (Illumina) and 2x250 paired-end cycles according to the manufacturer’s protocol.

### Sequence data analysis

Raw sequence reads were filtered using the MiSeq Sequencer System Software (Illumina) to remove low quality sequences and trimmed to remove adaptor sequences. Sequences passing the following criteria were used for further analysis.

### Genomic data

As described in the Illumina procedures guide, libraries were prepared with the Nextera™ XT DNA Library Prep kit.

(https://support.illumina.com/downloads/nextera_xt_sample_preparation_guide_15031942.html). Assembly, annotation and genome statistics were performed using the INNUca assembly pipeline (https://github.com/B-UMMI/INNUca), the RASTtk annotation tool (http://tutorial.theseed.org/services/docs/invocation/Iris/iris.html) and the QUAST quality assessment tool (http://quast.sourceforge.net/), respectively.

### Evaluation of the immunogenic potential

The 155,700 proteins of the 16 genomes analyzed were attributed an immunogenicity score using the VaxiJen® online software (http://www.ddg-pharmfac.net/vaxijen/VaxiJen/VaxiJen.html). As recommended by the software guidelines, a threshold value of 0.5 was used to categorize the identified proteins as probable antigens or non-antigens. For each *C. perfringens* genome analyzed, all proteins were sub-divided according to their VaxiJen® score. As most proteins showed scores between 0.5 and 0.9, a range of 0.2 was set to allow for a more refined description of the results. For scores above 0.9, two categories of scores were established and included proteins with scores between 0.9 and 1.5, as well as scores of 1.5 and above. Immunogenicity scores were therefore divided in four categories: 0.5–0.7, 0.7–0.9, 0.9–1.5, 1.5 and over. For each category, virulent strains were compared with one another in order to identify common proteins sharing a same annotation and a threshold of 90% similarity in the amino acid sequence.

### Protein localisation

The protein sub-cellular localisations were investigated using pSORTb (https://www.psort.org/psortb/), CELLO http://cello.life.nctu.edu.tw/, Gpos-mPLoc (http://www.csbio.sjtu.edu.cn/bioinf/Gpos-multi/) and FUEL-mLoc (http://bioinfo.eie.polyu.edu.hk/FUEL-mLoc/) software programs and a consensus decision-making approach was used to assign a final localisation status to each protein. In the case of ambiguous results (two distinct predicted localisations), both localisations were considered for the subsequent steps of the analysis. The presence of transmembrane regions and signal peptides was predicted using TMHMM® software (http://www.cbs.dtu.dk/services/TMHMM/) and SignalP® software (http://www.cbs.dtu.dk/services/SignalP/), respectively. In the absence of any signal peptide, the online software SecretomeP (http://www.cbs.dtu.dk/services/SecretomeP/) was used to assess the possibility of an alternate secretion pathway.

### Final putative antigenic protein selection

All candidate proteins were manually inspected to confirm they met the selection criteria: i) immunogenicity score above the 0.5 threshold value ii) amino acid sequence showing 90% similarity among all the analyzed virulent strains, iii) sequence either absent or different (below 90%) in amino acid composition when compared against the commensal *C. perfringens* studied. The presence of the candidate proteins identified was also analyzed in publicly available *C. perfringens* genomes through a BLAST search.

### Statistical analysis

Both the Student’s T-test for unequal variances and the Wilcoxon two-sample test were used to assess differences between virulent and commensal strains on the basis of assembly, localisation, and immunogenicity scores.

## Results

Table 2 presents the information pertaining to the 16 *C. perfringens* strains analyzed in this study. The profiles selected varied according to the strain type, the *netB* gene carriage, the PFGE profile and the type IV pilus profile. This table shows that six strains, from both the virulent and commensal types, were carrying the *netB* gene, leaving 10 strains with a negative *netB* profile. A total of seven different PFGE profiles and 12 distinct type IV pilus profiles represented the diversity among the *C. perfringens* strains analyzed in this study. On average, the genome length, the GC content, and the number of coding regions were all similar between the virulent and commensal strains assembled and statistical analyses revealed no significant differences for these results. Statistical analysis however showed that the genome assembly of commensal *C. perfringens* generated significantly (*p* = 0.043) less contigs (66) than the assembly of virulent strains (360).

As shown in Table 3, the sub-cellular localisation was predicted from the whole proteome of each strain. For both virulent and commensal types, cytoplasmic proteins were identified in higher proportions, followed by membrane proteins and by extracellular proteins. Statistics revealed that virulent strains had significantly (p = 0.018) more proteins than commensal *C. perfringens* strains. Furthermore, a higher number of extracellular (p < 0.0001) and cytoplasmic (p = 0.038) proteins was found in the virulent strains analyzed.

**Table 3:** Protein sub-cellular localisation predictions for virulent and commensal *C. perfringens* strains analyzed in this study.

Strain type	Strain identification	No. of proteins by localisation category			Total no. of proteins
		Extracellular	Cytoplasmic	Membrane	
Virulent	MLG_2313	436	2157	758	3351
	MLG_2514	483	1988	594	3065
	MLG_1202	521	2343	782	3646
	MLG_2915	514	2086	655	3255
	MLG_7814	470	2312	774	3556
	MLG_7820	498	2235	732	3465
	**AVERAGE**	**487**	**2187**	**716**	**3390**
Commensal	MLG_5806	301	1979	722	3002
	MLG_4206	325	2043	718	3086
	MLG_3406	410	1989	692	3091
	MLG_5213	422	2154	768	3344
	MLG_0612	417	2151	760	3328
	MLG_3119	358	2031	704	3093
	MLG_2919	327	2045	720	3092
	MLG_2719	341	2028	725	3094
	MLG_2019	315	1978	623	2916
	MLG_1619	346	1922	676	2944
	**AVERAGE**	**356**	**2032**	**711**	**3099**

Results presented in Table 4 show the total number of proteins in each genome predicted as antigenic by the VaxiJen® software (immunogenicity score ≥ 0.5) and the total number of proteins predicted as non-antigenic (immunogenicity score < 0.5). Although virulent *C. perfringens* strains revealed a higher mean number of proteins (3390) than the commensal strains (3099), both strain types appeared to be composed of a higher predicted number of non-antigenic proteins according to VaxiJen® (< 0.5). The number of proteins predicted as non-antigenic was not significantly different between both strain types. On average, virulent strains presented 1.6% more antigenic proteins than commensals. Statistical analysis showed that the number of probable antigens was significantly higher in virulent strains (p < 0.0001). Table 4 also shows that virulent *C. perfringens* were characterized by the presence of more probable antigenic proteins with a higher immunogenicity score. Statistical analysis revealed significant differences between virulent and commensal *C. perfringens*, where virulent strains were composed of more proteins with immunogenicity scores between 0.5 and 0.7 (p = 0.018) and high scores between 0.9 and 1.5 (p = 0.0025).

**Table 4:** Immunogenic potential of all proteins in genomes of virulent and commensal strains of *C. perfringens* analyzed

Strain type	Strain identification	No. of proteins/genome	% of genome identified as antigenic	No. of proteins as probable and improbable antigen		No. of proteins for each immunogenicity score category			
				Improbable antigen [score < 0,5]	Probable antigen [score > 0,5]	[0,5-0,7]	[0,7-0,9]	[0,9-1,5]	> 1,5
Virulent	MLG_2313	3351	33,24%	2237	1114	879	192	42	0
	MLG_2514	3065	34,26%	2015	1050	812	182	54	2
	MLG_1202	3646	34,20%	2399	1247	979	210	56	2
	MLG_2915	3255	33,79%	2155	1100	845	190	64	1
	MLG_7814	3556	34,06%	2345	1211	955	199	55	2
	MLG_7820	3465	33,68%	2298	1167	906	202	56	3
	**Average**	**3390**	**33,87%**	**2242**	**1148**	**896**	**196**	**55**	**2**
Commensal	MLG_5806	3002	32,31%	2032	970	788	146	35	1
	MLG_4206	3086	32,73%	2076	1010	819	149	42	0
	MLG_3406	3091	32,68%	2081	1010	817	147	46	0
	MLG_5213	3344	33,07%	2238	1106	883	181	42	0
	MLG_0612	3328	33,02%	2229	1099	883	176	40	0
	MLG_3119	3093	31,49%	2119	974	786	149	40	0
	MLG_2919	3092	31,47%	2119	973	784	148	41	0
	MLG_2719	3094	31,45%	2121	973	783	149	41	0
	MLG_2019	2916	32,30%	1974	942	763	741	38	0
	MLG_1619	2944	32,17%	1997	947	771	137	39	0
	**Average**	**3099**	**32,27%**	**2099**	**1000**	**808**	**212**	**40**	**0,1**

Table 5 presents the selection of 12 putative antigenic proteins and their respective description. This selection was based on the combination of the information pertaining to the immunogenicity score, the presence in virulent and absence from the commensal *C. perfringens* genomes analyzed and the predicted localisation. The predicted localisation for these candidates often showed ambiguity between the two prediction softwares used. For all selected candidates, immunogenicity scores varied between 0.529 and 1.515. The length of the selected proteins ranged from 87 amino acids up to 696 amino acids long. The number of transmembrane regions, the presence of a signal peptide and the alternative secretion pathway score further describe the proteins analyzed. For a specific candidate protein, transmembrane regions were either absent or present at most 3 times. Only four proteins revealed the presence of a signal peptide and 9 of the 12 candidate proteins showed a score above 0.9 for an alternative secretion pathway.

**Table 5:** Characteristics of the putative antigenic proteins identified from NE-causing *C. perfringens*

Protein identification	Protein length (aa.)	Predicted immunogenicity score	Predicted localisation	No. of TM regions	Signal P score	SecretomeP Score
P264–1	87	1.5149	Ex/Cy	–	–	0.947
P509	168	0.8671	Ex	–	–	0.947
P384	127	0.7736	CM	3	–	0.964
P216	72	0.7468	Cy/Ex	–	–	0.879
P1074	357	0.7339	CW/Ex	1	0.753	0.934
P153	50	0.7228	Ex/CM	–	–	0.948
P1569	522	0.7208	CW/CM	2	0.836	0.937
P804	267	0.7131	Ex/CM	1	–	0.929
P351	117	0.6004	Cy	–	–	0.082
P2091	696	0.6715	CM/CW	2	0.674	0.929
P561	186	0.634	Ex	–	–	0.929
P759	219	0.5258	CM/CW	1	0.626	0.856

Predicted localisation is described as Ex: extracellular, *Cy* Cytoplasmic, *CM* Cytoplasmic membrane, *CW* Cell wall. TM stands for transmembrane regions.

Table 6 presents a BLAST search comparing the proteins selected in this study and other publicly available proteins. Identity thresholds show results for perfect identity matches and results below a perfect match. From this table, we can observe that all selected proteins showed a 100% identity match to publicly available proteins of *C. perfringens* strains. For identity scores below 100%, proteins were found in various other bacterial species.

**Table 6:** Identification of proteins by BLAST analysis

Protein ID	BLAST (100% identity)		BLAST (identity <100%)	
	Protein match	Species	Protein match	Species
P264–1	Hypothetical protein	*C. perfringens*	Hypothetical protein	*C. ventriculi* 85% *C. tarantellae* 57% *C. sp. TW1* 75% *C. gasigenes* 56%
P509	Prepilin N-terminal cleavage methylation domain	Various	Type II secretion system protein	*T. thalassicus* 85%
P1074	Pilus backbone structural protein	*C. perfringens*	Hypothetical protein	*Blautia N6H1–15* 99% *C. scindens* 94%
P153	Hypothetical protein	*C. perfringens*	Hypothetical protein	*C. amazonitimonense* 98% *C. culturomicum* 96%
P2091	Cna B-type domain-containing protein	*C. perfringens*	Collagen adhesin precursor Cna B-type domain-containing protein	*S. dysgalactiae* 90% *S. agalactiae* 90%
P561	Prepilin N-terminal cleavage methylation domain	Various	Type II secretion system protein	*C. magnum* 98% *C. amylolyticum* 99%

## Discussion

### The comparative and subtractive reverse vaccinology approach

To date, alternatives to antimicrobials have shown to be partially efficient for keeping NE under control and the identification of specific *C. perfringens* antigens that could contribute to the development of a protective immunity in broiler chickens would greatly lower the burden of this disease in broiler chicken flocks raised without routine use of antibiotics. Using a comparative and subtractive reverse vaccinology approach, this study identified putative candidate proteins of NE-causing *C. perfringens* for which the role in NE pathogenesis and in the development of a protective immunity in broiler chickens exposed to NE-causing *C. perfringens* strains deserves more attention.

As opposed to a conventional comparative and subtractive reverse vaccinology approach, the methodology used in this study was based on a reverse selection process for the identification of the putative proteins, where the first step for selection was the predicted immunogenicity scores [15, 16]. Conventional comparative reverse vaccinology will typically suggest a first selection of the candidate proteins based on their sub-cellular localisation, assuming that the identified antigens imperatively need to be localized at the cell surface in order to be accessible to the immune system of the host [18]. The main advantage from adopting a reverse selection approach resides in the fact that every single protein, whatever its predicted localization, could be evaluated through the antigen prediction step, without any prior selection based on other characteristics or parameters. The approach used in this study allowed for the inclusion of proteins associated with cellular organelles, as well as multifunctional proteins with different domains and various secretion pathways. Thus, proteins classified in the larger sub-cellular localisation category and not only in the extracellular category were considered [18]. Moonlighting proteins are a good example of multifunctional proteins as they have been recognized to play one or multiple roles, such as cytokines, chaperones, adhesins and transmembrane channels [19]. It is therefore, considered that moonlighting proteins can potentially be part of the initial steps of an infection process, such as adhesion, emphasizing the importance of not dismissing these potential antigens when identifying putative candidates. Specifically, for *C. perfringens*, the glyceraldehyde-3-phosphate dehydrogenase (GAPDH) protein, which corresponds to the definition of a moonlighting protein, had previously been identified at the bacterial cell surface [20, 21]. Moreover, this glycolytic enzyme was previously associated with the adhesion and the virulence ability of other bacterial pathogens such as *Streptococcus pneumonia*e and *Staphylococcus aureus* [20, 22]. The moonlighting properties of the GAPDH protein include a glycolytic activity, as well as a contribution to the capacity of the bacteria to bind to fibronectin and plasminogen. With the absence of a clear secretion signal or of transmembrane regions, the presence of this protein at the surface of *C. perfringens* can be explained by a lysis of the bacterial cell and the subsequent release of the GAPDH which then binds to neighboring cells [21].

Results from this study are similar to those of previous reverse vaccinology studies in terms of the number of putative candidate proteins identified. Most of the reverse vaccinology studies conducted so far reported between 3 and 15 putative candidate antigens based on various selection criteria. However, as described earlier, these studies used a selection process based on protein localisation as the first step of the *in silico* analysis and they reported less than 50 proteins remaining after conducting the first step of the analysis due to the exclusive selection of extracellular or membrane proteins [15, 23, 24]. A reverse vaccinology study on *Leptospira interrogans* strains presented a similar pattern, however using a more complex method for selecting proteins, this method considering the predicted localisation, at least 3 softwares, and a consensus method to predict protein localisation [16]. The conventional reverse vaccinology approach assesses protein antigenic potential at different stages of candidate selection and scores usually vary greatly [15]. As Vaxijen® is one of the most used softwares for this part of methodology, most studies followed software guidelines and selected proteins with scores above 0.5 [18]. Few studies reported high immunogenicity scores with most scores ranging between 0.4 and 0.7 [15, 23]. For all the *C. perfringens* genomes analysed in the present study, we showed a high number of proteins in the same range [0.4–0.7], but also the presence of proteins with scores higher than 0.7. We also found 8 out of 12 selected proteins for which the predicted immunogenicity score was over 0.7, while the 4 other candidate proteins were identified with scores between 0.5 and 0.7. To our knowledge, this study is the first to report a proteome description using reverse vaccinology and also the first to describe the proteome of *C. perfringens* strains.

Even if the presence of transmembrane (TM) regions was routinely used as an assessment criterion in the reverse vaccinology approach, the importance of those regions in the selection of putative antigenic candidates has not been unanimous. A study showed that the presence of TM regions was a disqualifying factor in order to facilitate the expression of protein candidates in laboratory conditions [25]. Another study showed that the possibility to exclude the C-terminal region of TM domains facilitated the expression and purification of candidate proteins possessing these domains [26]. Therefore, based on bioinformatics analysis only, candidate proteins identified in the current study were not discarded on the basis of the presence of TM regions.

The number of probable antigens identified was higher in virulent *C. perfringens* than in their commensal counterpart, these probable antigens potentially being part of the typical genetic signature mainly associated with chromosomal and mobile genetic elements conferring a competitive advantage to virulent *C. perfringens* [27, 28]. Among these virulence factors, some have been recognized for their role in bacterial adhesion [27, 28]. The higher average number of predicted immunogenic proteins showing a score above 0.9 also supports a potential role of these putative proteins in the interaction of the NE-causing *C. perfringens* strains with its host. The analysis of virulent *C. perfringens* strains identified a higher number of extracellular proteins which could indicate the presence of virulence factors at the cell surface, further highlighting the need to better characterize the genetic factors and molecular mechanisms contributing to the virulence of NE-causing strains of *C. perfringens*. The surface localisation of some of these extracellular proteins that could play a role in the disease pathogenesis makes them even more interesting for their potential contribution to the first steps of NE pathogenesis [18].

### Candidate protein description

From the 12 selected proteins presented above, six were of higher interest based on their localisation, immunogenicity score and probable function. Therefore, proteins P264–1, P1074, P153, P209, P509 and P561 will be further described.

The candidate protein P264–1 that was attributed the highest VaxiJen® score (1.5149) was annotated as a hypothetical protein and was present in five of the six virulent strains. Although a 100% presence threshold in virulent strains was set, this protein was considered as a candidate due to its high immunogenicity score. As sequencing can be a factor in the observed variability, presence and absence from virulent or commensal strains, respectively, will be confirmed during ulterior PCR and sequencing analysis. The consensus approach used was not able to precisely localize this protein with certainty, as a dual prediction (extracellular and cytoplasmic) was obtained. The absence of a signal peptide and the high score predicted by the SecretomeP software could however indicate an alternative secretion pathway for this protein. The presence of multifunctional domains within this protein could explain the ambiguous results obtained from the localisation prediction step. A BLAST analysis of the sequence showed 100% similarity to a referenced hypothetical protein of *C. perfringens* (WP_057231569) and to other identical proteins from the publicly available Del1 and CP4 *C. perfringens* genomes. This candidate protein was also annotated as a hypothetical protein in other *Clostridium* species such as *C. ventriculi* and *C. gasigenes* for which 55 and 85% similarity levels were identified, respectively. To our knowledge, there is no additional information available on the identity and putative function of this hypothetical protein, neither on its role in the virulence of NE-causing *C. perfringens*.

The second candidate protein, P1074, showed a VaxiJen® score of 0.7339, was 357 amino acid long and was identified as both an extracellular and membrane hypothetical protein. This protein was present in all the virulent *C. perfringens* strains analyzed in this study and as for P264–1, a 100% similarity level with a known protein of *C. perfringens* (WP_057230739) was found. The TMHMM and SignalP analyses revealed the presence of both a signal peptide and a transmembrane region. BLAST results were in accordance with a Pfam search identifying the sequence of P1074 as part of the FctA family (PF12892), with its model protein Spy0128, a pilin protein of *Streptococcus pyogenes* encoded by the pilin backbone gene *spy0128*. An *in vivo* experiment showed a role of *S. pyogenes* pilin in adherence to host cells and in the formation of biofilms, all elements described in the pathogenesis of NE [29]. Based on these results, P1074 identified in virulent *C. perfringens* could potentially play a role in the adherence and colonisation ability of NE-causing strains of this bacterial species during the initial steps of the NE pathogenesis.

The 50 amino acid long candidate protein P153, with an immunogenicity score of 0.7228, showed 100% similarity with a previously reported hypothetical protein of *C. perfringens* (BAB80098.1). The analysis of this protein also showed the absence of a signal peptide or a transmembrane region. The SecretomeP score (0.948), above the threshold, predicted for this protein suggests an alternative secretion pathway which could translate in a moonlighting ability for this candidate. A comprehensive literature search did not yield any further information regarding the function, potential role in virulence or immunogenic properties for this protein. This putative antigenic protein was also present as a hypothetical protein in the publicly available genomes of NE-causing *C. perfringens* CP15 and LLY_N11 strains, with a 100% identity score. Further BLAST investigations showed that this hypothetical protein was present in other *Clostridium* species such as *C. amazonitimonense* and *C. culturomicum*, but no further information on its function was available as it also was annotated as a hypothetical protein.

P2091, which was identified by the current study as a hypothetical protein with an immunogenicity score of 0.6715, was predicted to be localized at both the cell-wall and bacterial membrane level. When searching the Pfam database, a perfect match with the PF05737 protein family associated with collagen binding was found. A BLAST search confirmed this result as P2091 from the current study matched (90%) similar proteins in other bacterial genomes such as *Streptococcus dysgalactiae* and *Streptococcus agalactiae*. From all the bacterial genomes screened in the National Center for Biotechnology Information (NCBI) public database, this protein was associated with the B-type domain of the Cna protein which is a surface protein mediating collagen binding [30]. The *cnaA* gene has been associated with adhesion to type IV and V collagen and a correlation between the presence of the gene in some *C. perfringens* strains and the capacity of adhesion to collagen has been demonstrated [31]. Moreover, the *cnaA* gene has been identified in NE causing *C. perfringens* strains, with both positive and negative *netB* profiles [12, 31]. The results obtained in the current study suggest similar conclusions as the *cnaA* gene was present in the six virulent and two of the ten commensal strains analyzed, regardless of their *netB* status.

As previously mentioned, protein P264–1, showing the highest immunogenicity score, was absent from all the commensal *C. perfringens* genomes analyzed in the current study. In comparison, the three proteins described above (P1074, P153 and P2091) were absent from all, except two of the commensal *C. perfringens* strains analysed. Although their absence from commensal strains was a selection criterion, these proteins were considered as potential candidates based on the other selection criteria mentioned previously and also based on their suspected functions in adherence and colonization. As well as for P264–1, sequencing can be a factor explaining the observed sequence variability, and the presence and absence from virulent and commensal strains, respectively, will be verified using an alternative approach such as PCR and sequencing. Genetic similarities between commensal and virulent strains of *C. perfringens* could however indicate the sharing of some genetic material, a feature recognized as a major contributing element in the shaping of the populations of this bacterial species [32]. It could also be hypothesized that the virulent strains analyzed in this study were more competitive; therefore, limiting the disease potential of other strains, despite those genetic similarities. Indeed, it has been shown that during a NE outbreak, a particular clone dominates over the other *C. perfringens* strains present in the same population until the disease outbreak resolves and some diversity is observable again in the *C. perfringens* population [33]. Even though the commensal strains analyzed in this study were isolated from flocks affected with NE, they were not identified as clones causing the disease outbreak but were rather representatives of the *C. perfringens* population diversity after the disease outbreak [5]. It is therefore possible that the genome of the commensal strains studied harbored some putative virulence factors contributing to a fitness advantage, though not sufficient to confer these strains a true disease-causing ability [7, 11, 27].

Two additional hypothetical proteins, P509 and P561, were selected based on their extracellular localisation, their immunogenicity score above 0.6, and their suspected function as part of a pilin domain, potentially playing a role in *C. perfringens* colonization. Using a BLAST search approach on the sequences encoding both candidate proteins, the analysis revealed a 100% similarity level with known *C. perfringens* N-terminal cleavage and methylation domains of prepilin-type proteins. When performing a Pfam search, both proteins were associated with the N-Methyl protein family PF07963 known for its short motif and its role in the methylation of the phenylalanine residue at the N-terminal domain of the pilin proteins. This domain has previously been reported to be part of the toxin-coregulated pilus subunit TcpA identified in pathogenic *Vibrio cholerae* and representing one of the virulence factors involved in the colonization ability of this pathogen [34].

## Conclusion

In the context of reducing the global use of antibiotics in animal production, a better understanding of the genetic features of NE-causing *C. perfringens* is crucial, since it could contribute to the development of a protective immunity in exposed broiler chickens, as well as to the search for alternative control methods that will help keep this animal disease under control. The aim of this study was to identify, using an *in silico* analysis approach involving comparative and subtractive reverse vaccinology, putative antigenic proteins that could contribute to the development of a protective immunity in exposed broiler chickens. The immunogenicity score, the protein localisation prediction and presence solely in virulent strains were the main criteria used to select the candidate proteins in the comparative and subtractive reverse vaccinology approach proposed in the current study. A total of 12 different candidates were presented, with some showing similarity with known proteins or protein families for which a role in host cell colonization has been identified in other bacterial pathogens. Further investigations on these candidate proteins will help clarify their functions, roles in disease pathogenesis and antigenic potential, shedding completely new light on *C. perfringens* virulence and contributing to a better understanding of NE pathogenesis.

## Data Availability

The datasets generated and/or analyzed during the current study are publicly available in the SRA database as project ID: PRJNA734442.
